# Alleviating effect of quercetin on cadmium-induced oxidative damage and apoptosis by activating the Nrf2-keap1 pathway in BRL-3A cells

**DOI:** 10.3389/fphar.2022.969892

**Published:** 2022-08-11

**Authors:** Jicang Wang, Ke Wang, Lulu Ding, Pengli Zhao, Cai Zhang, Hongwei Wang, Zijun Yang, Zongping Liu

**Affiliations:** ^1^ College of Animal Science and Technology, Henan University of Science and Technology, Luoyang, China; ^2^ College of Veterinary Medicine, Yangzhou University, Yangzhou, China

**Keywords:** cadmium, quercetin, BRL-3A cells, apoptosis, oxidative damage, Nrf2-Keap1 pathway

## Abstract

Cadmium (Cd) is a toxic heavy metal extensively used in industrial and agricultural production. Among the main mechanisms of Cd-induced liver damage is oxidative stress. Quercetin (QE) is a natural antioxidant. Herein, the protective effect of QE on Cd-induced hepatocyte injury was investigated. BRL-3A cells were treated with 12.5 μmol/L CdCl_2_ and/or 5 μmol/L QE for 24 h. The cells and medium supernatant were collected, and the ALT, AST, and LDH contents of the medium supernatant were detected. The activities or contents of SOD, CAT, GSH, and MDA in cells were determined. Intracellular ROS levels were examined by flow cytometry. Apoptosis rate and mitochondrial-membrane potential (ΔΨm) were detected by Hoechst 33,258 and JC-1 methods, respectively. The mRNA and protein expression levels of Nrf2, NQO1, Keap1, CytC, caspase-9, caspase-3, Bax, and Bcl-2 were determined by real-time PCR (RT-PCR) and Western blot methods. Results showed that Cd exposure injured BRL-3A cells, the activity of antioxidant enzymes decreased and the cell ROS level increased, whereas the ΔΨm decreased, and the expression of apoptotic genes increased. Cd inhibited the Nrf2-Keap1 pathway, decreased Nrf2 and NQO1, or increased Keap1 mRNA and protein expression. Through the combined action of Cd and QE, QE activated the Nrf2-Keap1 pathway. Consequently, antioxidant-enzyme activity decreased, cellular ROS level decreased, ΔΨm increased, Cd-induced BRL-3A cell damage was alleviated, and cell apoptosis was inhibited. After the combined action of QE and Cd, Nrf2 and NQO1 mRNA and protein expression increased, Keap1 mRNA and protein expression decreased. Therefore, QE exerted an antioxidant effect by activating the Nrf2-Keap1 pathway in BRL-3A cells.

## Introduction

Cadmium (Cd) is one of the most toxic pollutants in natural and occupational environments. Industrial heavy-metal pollution, fossil-fuel use, and other human activities affect the existence of Cd (Liu et al., 2014). Cd is also extensively used in electroplating, painting, anticorrosion agents, plastic stabilizers, and electrical contact materials. Once absorbed by the body, Cd persists in humans and animals for a long time, affecting various organs, including kidney, heart, liver, and brain (Winiarska-Mieczan et al., 2013; Amamou et al., 2015; Wen et al., 2021; Gong et al., 2022). The liver is the primary target organ for Cd to accumulate and exert its harmful effects. Most studies have shown that Cd is preferentially localized to hepatocytes *in vivo* and *in vitro* (Li et al., 2013; Horiguchi and Oguma, 2016).

Cd exposure induces oxidative stress in cells, leading to the accumulation of reactive oxygen species (ROS) and malondialdehyde (MDA), resulting in lipid peroxidation and oxidative damage (Vicente-Sánchez et al., 2008; Khan et al., 2019). Cd further causes changes in mitochondrial membrane permeability, releases CytC, and induces mitochondrial caspase-dependent apoptotic protein expression through a cascade reaction, leading to apoptosis (Banik et al., 2019).

Quercetin (QE), a natural flavonoid widely distributed and abundant in vegetables and fruits (Donmez et al., 2019), has strong antioxidant activity. It can directly scavenge reactive oxygen species, chelate metal ions, and inhibit oxidative damage. It also has anti-inflammatory, antitumor, antivirus, and liver- and cardiovascular-protective effects (Boots et al., 2008; Zhao et al., 2021).

The present study aimed to examine the impact of QE on Cd-induced apoptosis and oxidative damage in rat liver cells, as well as the role of the caspase-dependent and Nrf2 signaling pathways in these processes.

## Materials and methods

### Chemicals

Cadmium chloride (CdCl_2_; purity >99.99%) was purchased from Sigma–Aldrich Industrial Corporation. QE (purity >97%) was purchased from Shanghai Yien Chemical Technology (Shanghai, China). ALT, AST, and LDH kits were purchased from Nanjing Jiancheng Bioengineering Institute (Nanjing, China). Rat SOD, CAT, GSH, and MDA ELISA kits were purchased from Shanghai Yubo Biotechnology (Shanghai, China). Fetal bovine serum (FBS) was purchased from Thermo Fisher Scientific CN, Benzyl penicillin and streptomycin were purchased from Beyotime Biotechnology (Shanghai, China). β-Actin, CytC, caspase-9, caspase-3, Bcl-2, Bax, Nrf2, NQO1, and Keap1 were purchased from Proteintech Group (Wuhan, China). Detection kits of ROS, Hoechst 33,258, and Annexin V-FITC apoptosis were purchased from Beyotime Biotechnology (Shanghai, China). RNA-Isolation Total RNA Extraction Reagent, HiScript III RT SuperMix for qPCR (+gDNA wiper), and ChamQ universal SYBR qPCR Master Mix were purchased from novozan Biotechnology (Nanjing, China). All other routine chemicals and solvents were of pure analytical grade.

### Cell culture

BRL-3A cells were obtained from Yangzhou University. They were cultivated in high-glucose DMSO containing 10% FBS, 2% streptomycin, and penicillin. They were then cultured in an incubator at 37°C with 5% CO_2_. The cells were divided into four groups and treated in medium supplemented with CdCl_2_ and/or QE for 24 h. The treatment results are shown in Table 1.

**TABLE 1:** Treatment of cells.

Group	Treatment
Control group	normal medium
Cd group	12.5 μmol/L CdCl_2_
Cd + QE group	12.5 μmol/L CdCl_2_+5 μmol/L QE
QE group	5 μmol/L QE

### Cell viability detected by MTT

The BRL-3A cells were inoculated on 96-well plates with a cell density of 1 × 10^4^/L, and 200 μl culture medium was added to each well until the cell-coverage area reached 60–70%. Each well was exposed to 10 μl of MTT (5 mg/ml) after various treatments were conducted according to the experimental requirements. After gentle mixing and culturing at 37°C with 5% CO_2_ for 4 h, we carefully sucked and discarded the culture medium in the hole with a syringe. Then, 100 μl of formazan solubilization solution was added to each well. The 96-well plate was placed on a horizontal shaking table and shook slowly for 10 min. Absorbance was measured at 570 nm by enzyme immunoassay.

### Determination of liver-marker enzymes and antioxidants

ALT, AST, and LDH were measured using diagnostic kits. SOD, CAT, GSH, and MDA activities or content were measured using diagnostic ELISA kits following the manufacturer’s protocol.

### Intracellular ROS determination

The BRL-3A cells were inoculated in a six-well culture dish until the cell-coverage area reached 60–70%. Cells were then treated according to the experimental groups. After 24 h, the cells were incubated with the ROS detection kit. The cells were incubated in a 37°C cell incubator for 20 min. ROS level was measured by flow cytometry.

### Mitochondrial membrane potential (ΔΨm) detection

The BRL-3A cells were inoculated in six-well plates, and cells were treated according to experimental groups. The cells were incubated with the ΔΨm detection kit (JC-1) and then incubated again in a 37°C cell incubator for 20 min. According to the manufacturer’s protocol, changes in ΔΨm were detected.

### Hoechst 33258

Apoptosis was detected with a Hoechst 33258 kit. The BRL-3A cells were inoculated into six-well plates for 12 h and then treated with Cd (12.5 μmol/L) and QE (5 μmol/L) for 24 h. Following a PBS wash, cells were incubated for 30 min with Hoechst 33,258 staining solution in a cell incubator. The staining solution was discarded, and the cells were washed twice with PBS. An inverted fluorescence microscope was used to observe and photograph the cells.

### mRNA expression

Total RNA was extracted from rat liver tissue by using an RNA-isolater Total RNA Extraction Reagent. Then, using HiScript III RT SuperMix for qPCR (+gDNA wiper), total RNA was reverse transcribed to synthesize cDNA. We identified the mRNA sequence of rat caspase-9, caspase-3, and other genes from GenBank. The primers were designed using Primer Premier six and tested specifically in NCBI-Primer. Following the kit, ChamQ universal SYBR qPCR Master Mix was used for qRT-PCR. Three technical repetitions were performed for each sample, and the mean was considered to represent mRNA levels. β-Actin served as the endogenous control. The relative mRNA levels were analyzed by 2^−△△Ct^ method. The primers for Nrf2, Keap1, NQO1, Bcl-2, Bax, CytC, caspase-9, caspase-3, and β-actin are listed in Table 2.

**TABLE 2:** Gene primers sequence and their GenBank accession number used for qRCR.

Gene name	Accession number	Primer sequences (5′-3′)
Nrf2	NM_001399173.1	Forward: AGCACATCCAGACAGACACCA
		Reverse: TATCCAGGGCAAGCGACTC
Keap1	NM_057152.2	Forward: AGCAGGCTTTTGGCATCAT
		Reverse: CCGTGTAGGCGAACTCAATTAG
NQO1	NM_017000.3	Forward: GGTGAGAAGAGCCCTGATTGT
		Reverse: CTCCCCTGTGATGTCGTTTC
Bcl-2	NM_016993.2	Forward: CAAGCCGGGAGAACAGGGTA
		Reverse: CCCACCGAACTCAAAGAAGGC
Bax	NM_017059.2	Forward: CCGAGAGGTCTTCTTCCGTGTG
		Reverse: GCCTCAGCCCATCTTCTTCCA
Caspase-3	NM_012922.2	Forward: GCAGCAGCCTCAAATTGTTGACTA
		Reverse: TGCTCCGGCTCAAACCATC
Caspase-9	NM_031632.2	Forward: CTGAGCCAGATGCTGTCCCATA
		Reverse: CCAAGGTCTCGATGTACCAGGAA
CytC	NM_012839.2	Forward: GGAGAGGATACCCTGATGGA
		Reverse: GTCTGCCCTTTCTCCCTTCT
β-actin	NM_031144.3	Forward: AGGGAAATCGTGCGTGACAT
		Reverse: CCTCGGGGCATCGGAA

### Western blot

Following gentle washing with PBS, the cells were lysed with RIPA to extract total protein, and the cells were lysed with nuclear protein extract to extract nuclear protein. A BCA kit was used to extract and detect the protein concentration of each group. After adding loading buffer to the protein and boiling it for storage, SDS–PAGE electrophoresis (10% separation gel and 5% concentrated gel for protein electrophoresis) was performed. After transferring the protein onto PVDF membranes, they were sealed with 5% skimmed milk for 1 h. The primary antibody was cultured in a shaking table at 4°C for 12 h and washed with TBST. The secondary antibody was incubated for 1 h at room temperature and subjected to ECL chemiluminescence analysis with photos taken.

### Statistical analysis

SPSS (version 17) was used to analyze the data obtained under different experimental conditions. One-way ANOVA was used to compare results between the control and test groups.

## Results

### Effects of Cd and QE on cell viability in BRL-3A cells

**FIGURE 1:**
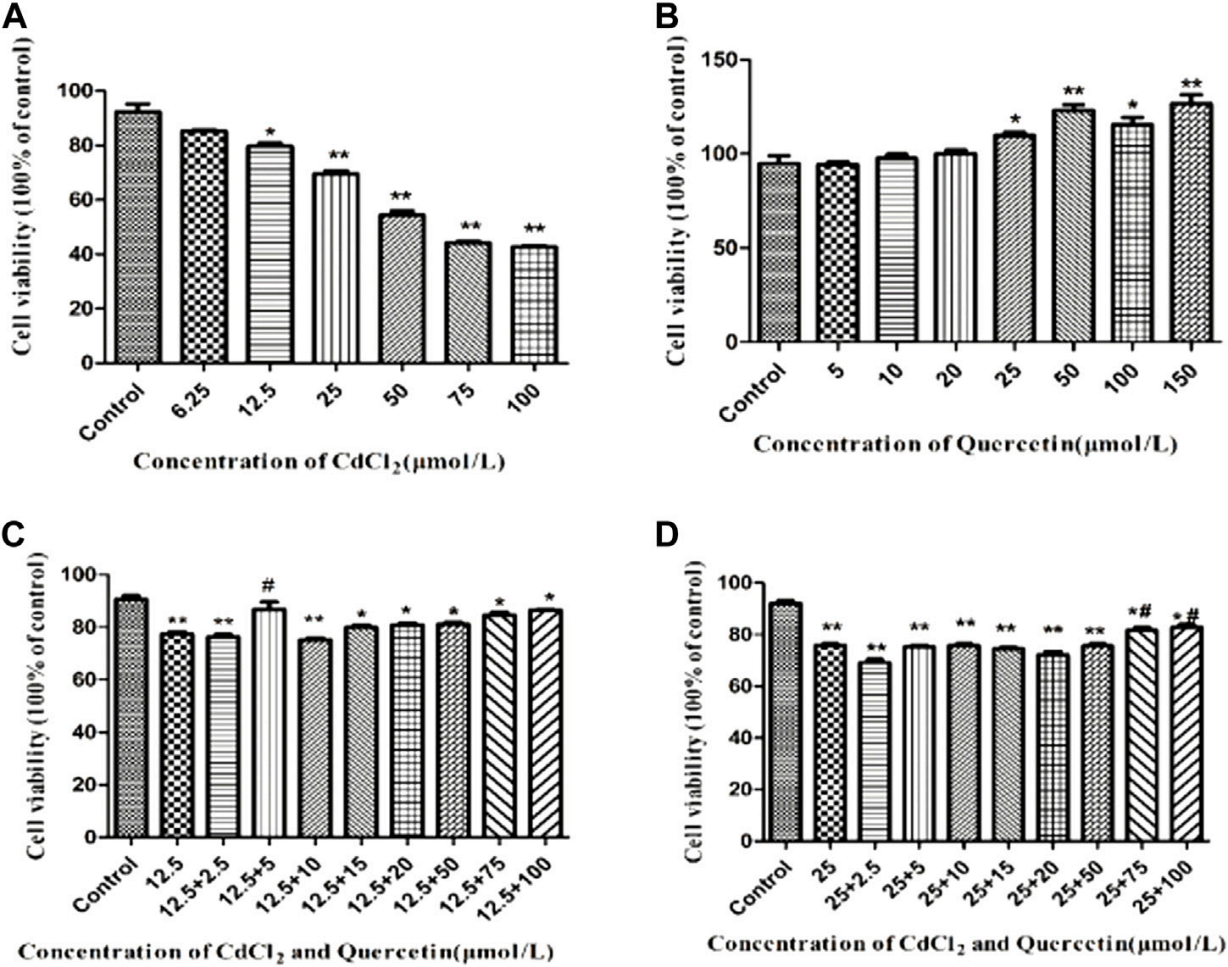
Detection of cell viability by MTT assay. The data are expressed in the form of (mean ± SEM), “*” indicates significant difference compared with the control group (*p* < 0.05), “**” indicates extremely significant difference compared with the control group (*p* < 0.01), “#” indicates significant difference compared with the 12.5 μmol/L CdCl_2_ group or the 25 μmol/L CdCl_2_ group (*p* < 0.05), “##” indicates extremely significant difference compared with the 12.5 μmol/L CdCl_2_ group or the 25 μmol/L CdCl_2_ group (*p* < 0.01).

BRL-3A cell viability was detected at six CdCl_2_ concentrations by MTT assay. As shown in Figure 1A, the IC_50_ of CdCl_2_ was about 50 μmol/L. Figure 1B shows that compared with the control group, when the QE concentration was 5–150 μmol/L, QE had no inhibitory effect on cell activity. According to the results in Figures 1A–D and referring to the findings of Yang et al. (2019), the CdCl_2_ and QE concentrations were finally determined to be 12.5 and 5 μmol/L, respectively, *in vitro*.

### Medium supernatant liver-marker enzyme status

**FIGURE 2:**
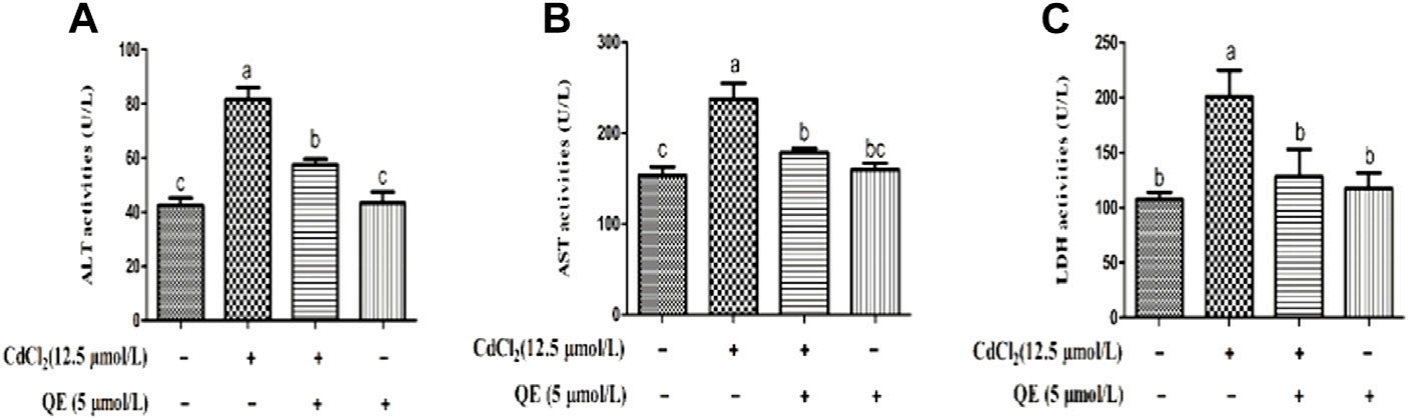
Effects of QE on Cd-induced changes in the levels of AST, ALT, and LDH in BRL-3A cells. The activities of ALT **(A)**, AST **(B)**, and LDH **(C)** in the medium supernatant were measured. Data are expressed as the mean ± SEM, and data with different letters (a, b, and c) represent significant differences (*p* < 0.05) among different treatments.

Figure 2 shows that after the CdCl_2_ treatment of BRL-3A cells for 24 h, the AST, ALT, and LDH contents in the medium of the Cd group significantly increased (*p* < 0.05). This finding indicated that the cell membrane was ruptured and liver enzymes were released. However, after cotreatment with QE, compared with the Cd group, the AST, ALT, and LDH contents in the medium decreased significantly (*p* < 0.05). This finding indicated that QE significantly improved the damage inflicted by CdCl_2_ to cells.

### Antioxidant-enzyme activity and oxidative injuries

**FIGURE 3:**
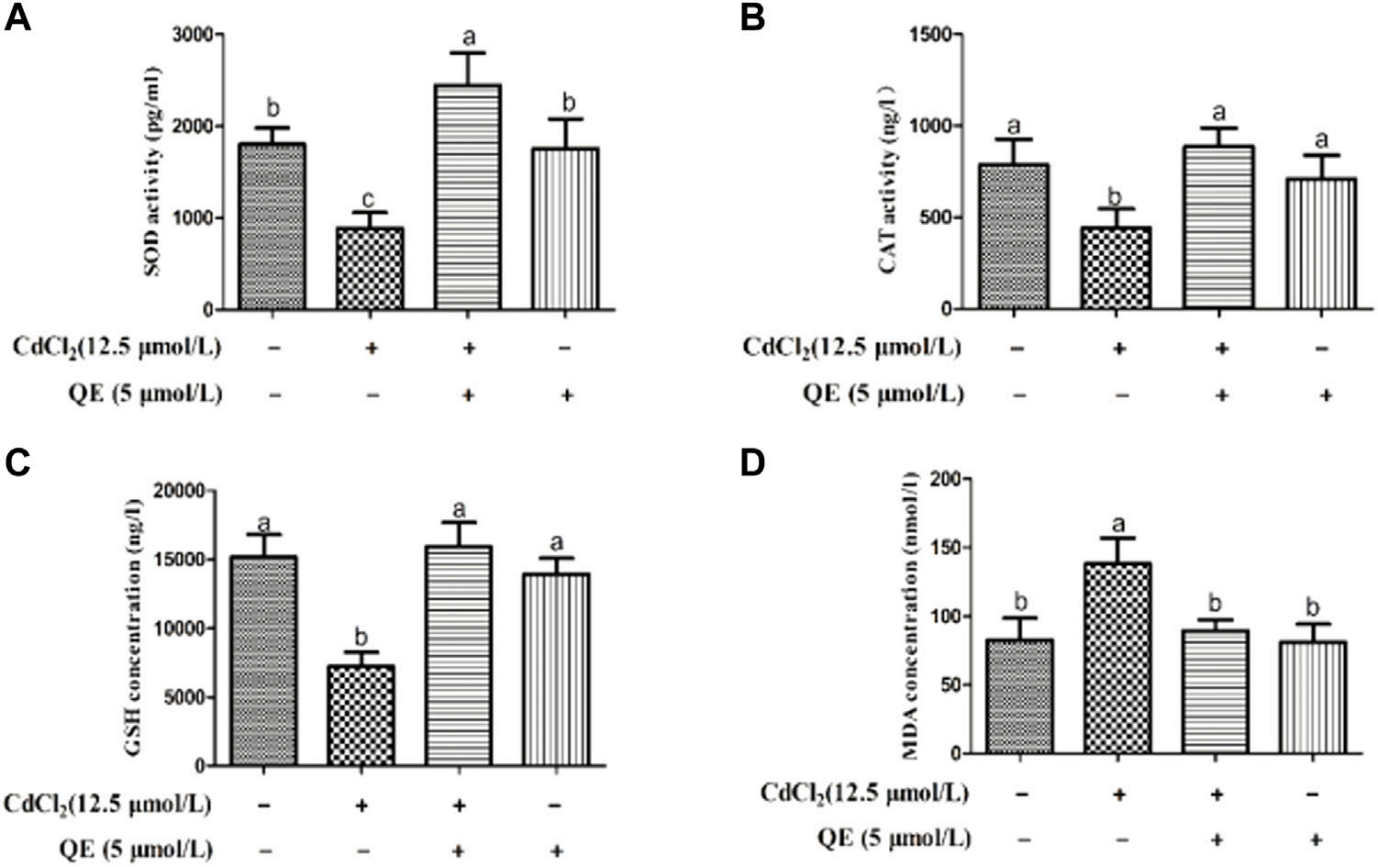
Effects of QE on cadmium-induced activities or contents of SOD, CAT, GSH, and MDA in BRL-3A cells. The SOD **(A)** and CAT **(B)** activities and the GSH **(C)** and MDA **(D)** concentrations in the cell supernatant were measured. Data are expressed as the mean ± SEM. Data with different letters (a, b, and c) represent significant differences (*p* < 0.05) among different treatments.

Figure 3 shows that compared with the control group, the activities of SOD and CAT in the Cd group decreased significantly, the GSH content decreased significantly, and the MDA content increased significantly (*p* < 0.05). After cotreatment with QE, compared with those in the Cd group, the SOD and CAT activities increased, the GSH content increased, and the MDA content decreased in the Cd + QE group, with significant differences (*p* < 0.05). Compared with those in the control group, the SOD and CAT activities and the GSH and MDA contents did not significantly differ (*p* > 0.05), and the levels returned to normal.

### Effects of QE on the mRNA Expression Levels of Cd-induced the Nrf2 Signaling Pathway in BRL-3A Cells

**FIGURE 4:**
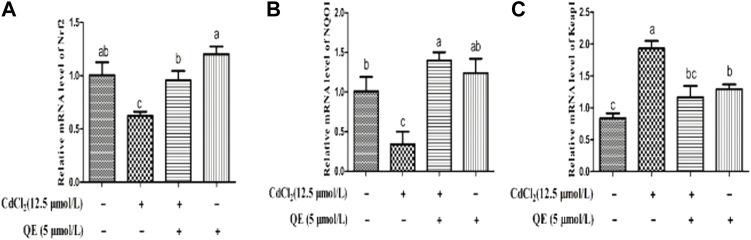
Effects of QE on the mRNA expression of Cd-induced the Nrf2 signal pathway related genes in BRL-3A cells. Relative mRNA levels of Nrf2 **(A)**, NQO1 **(B)**, and Keap1 **(C)** in BRL-3A cells. Data are expressed as the mean ± SEM, and data with different letters (a, b, and c) represent significant differences (*p* < 0.05) among different treatments.

Figure 4 shows that compared with the control group, the mRNA expression levels of Nrf2 and NQO1 in the Cd group decreased significantly (*p* < 0.05). The mRNA expression level of Keap1 increased significantly (*p* < 0.05). After cotreatment with QE, compared with the Cd group, the mRNA expression levels of Nrf2 and NQO1 in the Cd + QE group increased significantly (*p* < 0.05), whereas the mRNA expression level of Keap1 decreased significantly (*p* < 0.05), and the expression level tended to the control group.

**FIGURE 5:**
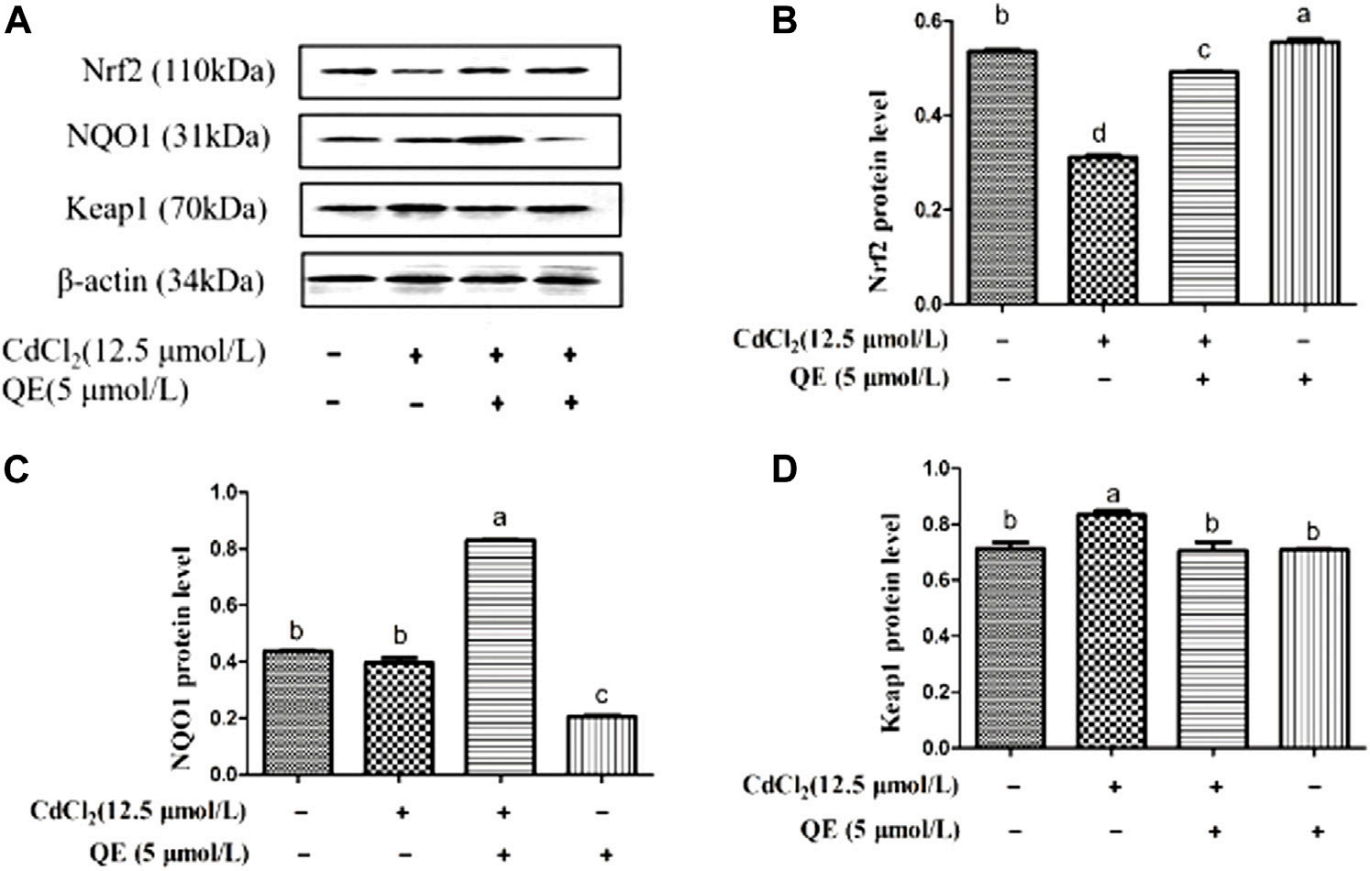
Effects of QE on protein expression of Cd-induced the Nrf2 signal pathway related genes in BRL-3A cells. The levels of Nrf2, NQO1 and Keap1 proteins **(A)** were measured by Western blot. The protein levels of Nrf2 **(B)** NQO1 **(C)** and Keap1 **(D)** in BRL-3A cells. β-actin was used as a control. The data are expressed in the form of mean ± SEM, and data with different letters (a, b, and c) represent significantly different (*p* < 0.05) among different treatments.

Figure 5A shows the protein-band diagram and the protein expression levels of Nrf2 signal pathway in BRL-3A cells. Compared with the control group, the expression level of Nrf2 protein in the Cd group decreased significantly (*p* < 0.05), whereas those of NQO1 protein decreased and Keap1 protein increased significantly. After QE treatment, compared with the Cd group, the protein expression levels of Nrf2 and NQO1 in the Cd + QE group increased significantly (*p* < 0.05), and the mRNA expression level of Keap1 decreased significantly (*p* < 0.05).

### Effects of QE on ROS level in Cd-induced BRL-3A cells

**FIGURE 6:**
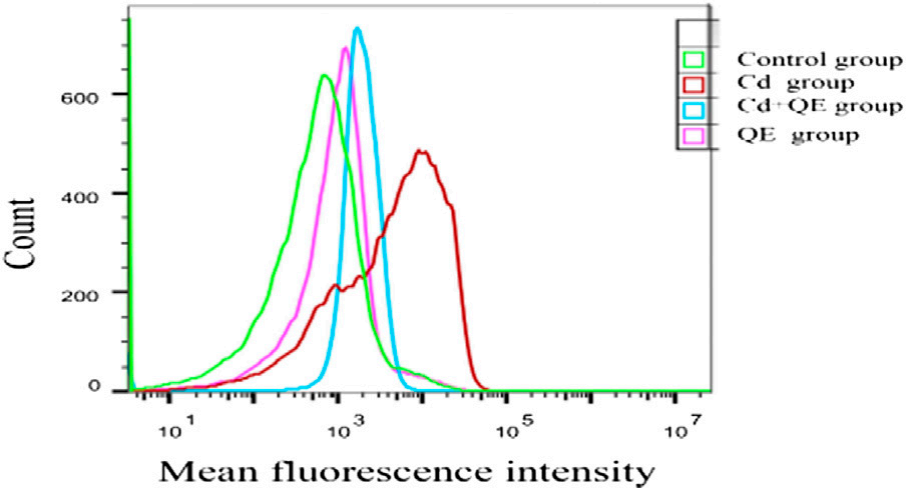
Effects of QE on ROS level in Cd-induced BRL-3A cells.

DCFH-DA is a fluorescent dye that can measure the activity of intracellular ROS, which can oxidize DCFH to generate fluorescent DCF. Figure 6 shows that when BRL-3A cells were treated with CdCl_2_, the fluorescence peak of the Cd group shifted to the right compared with the control group, indicating increased ROS generation. After QE intervention, compared with the Cd group, the fluorescence peak shifted to the left, indicating that QE reduced the ROS generation.

### Effects of QE on ΔΨm in Cd-induced BRL-3A cells

**FIGURE 7:**
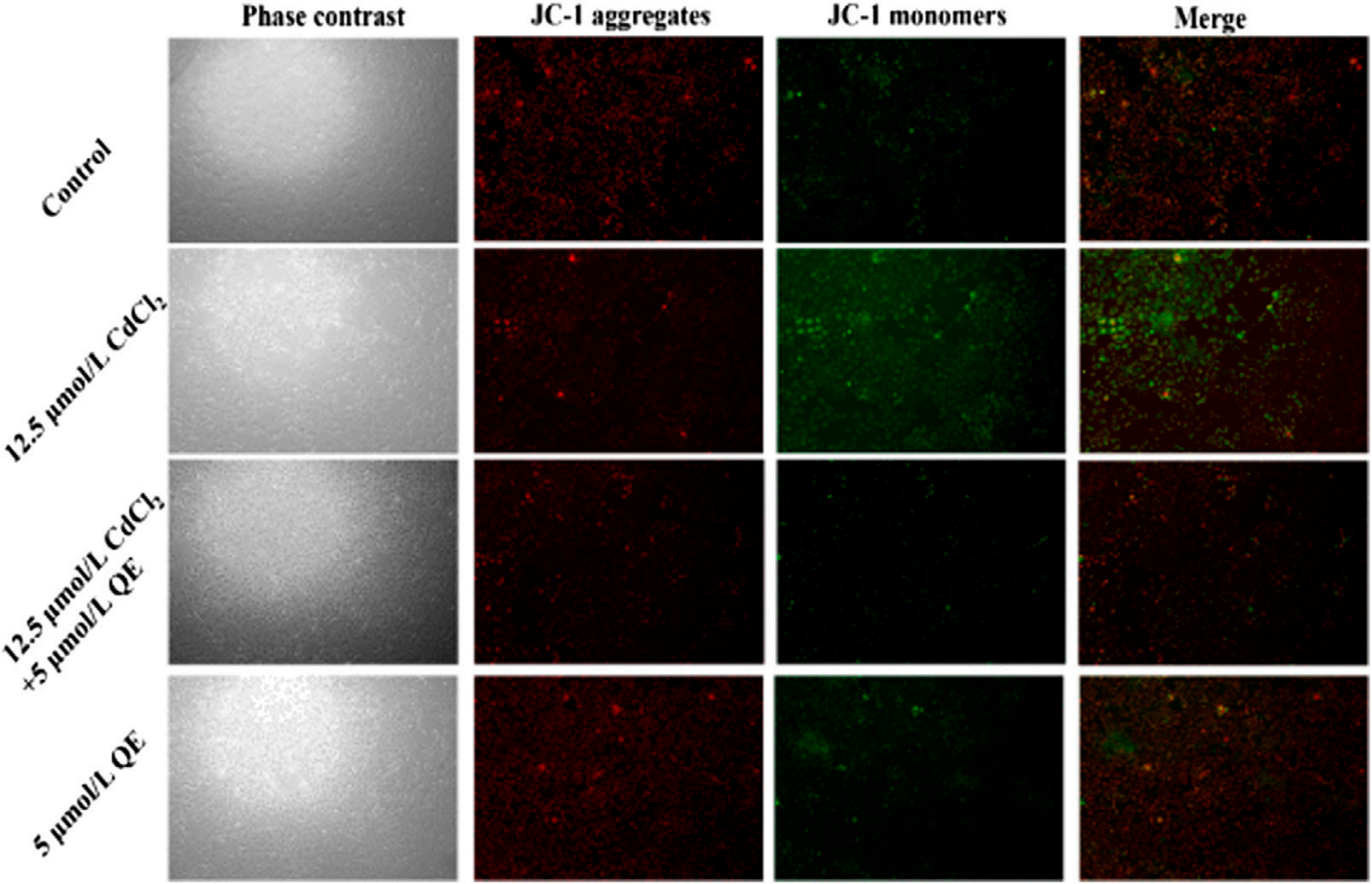
Effects of QE on mitochondrial-membrane potential in Cd-induced BRL-3A cells.

JC-1 (C_25_H_27_Cl_4_IN_4_) is a fluorescent probe used to detect ΔΨm. Red fluorescence indicates that the ΔΨm is relatively normal, and green fluorescence indicates decreased ΔΨm and possibly early-stage cell apoptosis. Figure 7 shows that cells in the control group were closely arranged under a microscope, grew well, and were oval and full. Under fluorescent conditions, the red fluorescence was strong, the green fluorescence was weak, and the ΔΨm is normal. Compared with the control group, cells in the QE group showed no difference in cell state and fluorescence intensity. Cells in the Cd group were long spindle shaped with sparse cells, and some cells died and fell off the bottom of the culture dish. The red fluorescence was weak and the green fluorescence was strong, and the ΔΨm decreased. Compared with the Cd group, cells were in good condition, the red fluorescence was enhanced, and the green fluorescence was weakened in the Cd + QE group. This finding indicated that QE could significantly improve the effect of Cd-induced BRL-3A cells on ΔΨm.

### Effects of QE on the mRNA Expression Levels of Cd-induced Apoptosis Genes in BRL-3A cells

**FIGURE 8:**
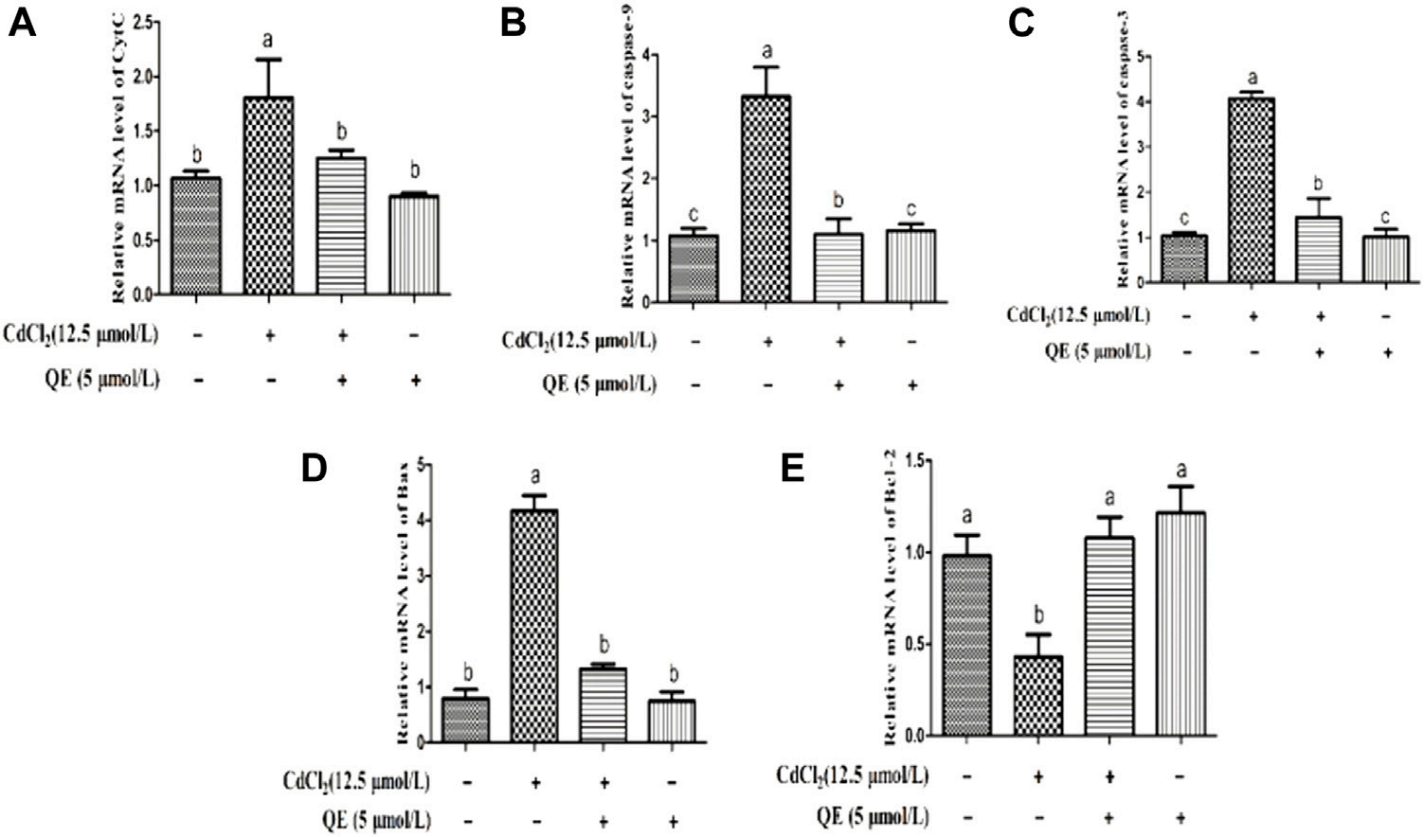
Effects of QE on Cd-induced BRL-3A cell apoptosis-related gene mRNA expression. The relative mRNA level of CytC **(A)**, caspase-9 **(B)**, caspase-3 **(C)**, Bax **(D)** and Bcl-2 **(E)** in BRL-3A cells. The data are expressed in the form of mean ± SEM, and data with different letters (a, b, and c) represent significantly different (*p* < 0.05) among different treatments.

Figure 8 shows that compared with the control group, the mRNA expression levels of CytC, caspase-9, caspase-3 and Bax in the Cd group increased significantly (*p* < 0.05), and the mRNA expression level of Bcl-2 in the Cd group decreased significantly (*p* < 0.05). After adding QE, the mRNA expression levels of CytC, caspase-9, caspase-3, and Bax in the Cd + QE group decreased significantly (*p* < 0.05), whereas the mRNA expression level of Bcl-2 increased significantly (*p* < 0.05) compared with the Cd group.

### Effects of QE on the Protein Expression Levels of Cd-induced Apoptosis Genes in BRL-3A Cells

**FIGURE 9:**
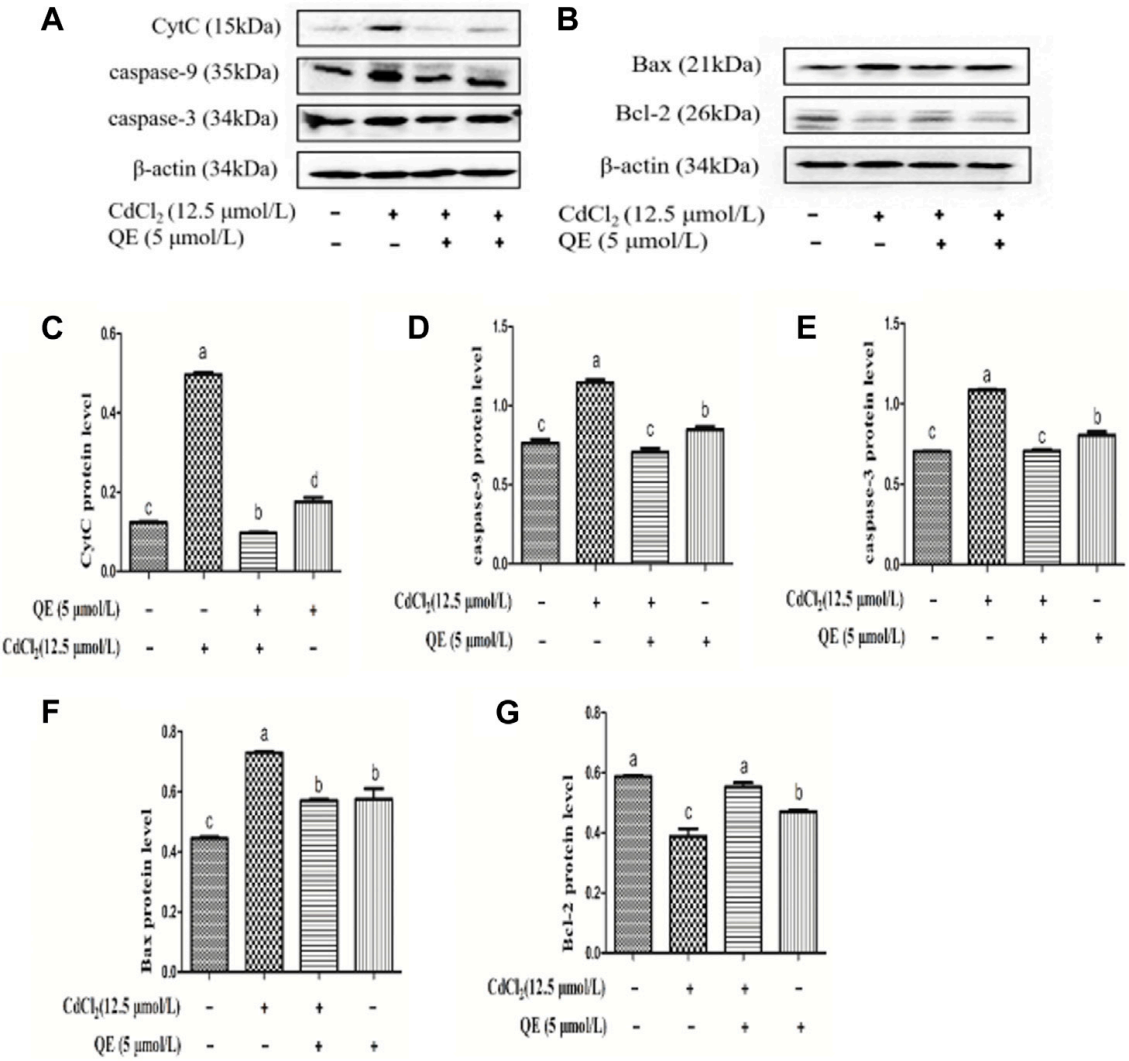
Effects of QE on Cd-induced BRL-3A cell apoptosis-related gene and protein expression. The levels of CytC, caspase-9, caspase-3, Bax and Bcl-2 proteins **(A-B)** were measured by Western blot. The protein levels of CytC **(C)**, caspase-9 **(D)**, caspase-3 **(E)**, Bax **(F)** and Bcl-2 **(G)** in BRL-3A cells. The data are expressed in the form of mean ± SEM, and data with different letters (a, b, and c) represent significantly different (*p* < 0.05) among different treatments.

Figure 9 shows the protein bands and protein expression levels of caspase-pathway-related genes. Compared with the control group, the protein expression levels of CytC, caspase-9, caspase-3, and Bax in the Cd group increased significantly (*p* < 0.05). The protein expression level of Bcl-2 in the Cd group decreased significantly (*p* < 0.05). After adding QE, compared with the Cd group, the protein expression levels of CytC, caspase-9, caspase-3, and Bax in the Cd + QE group decreased significantly (*p* < 0.05), and whereas protein expression level of Bcl-2 increased significantly (*p* < 0.05).

## Discussion

Cd is a toxic heavy metal widely used in industrial and agricultural production. The applications of Cd cause environmental pollution that is becoming increasingly serious. Cd can inflict serious toxic damage to the liver, kidney, and reproductive system of humans and animals. The liver is the main target organ of Cd poisoning, and its damage is serious (Gong et al., 2019). Increasing evidence indicates that Cd accumulates in the liver, induces oxidative stress and apoptosis, and leads to liver damage (Cao et al., 2017). Oxidative stress and apoptosis can be inhibited by regulating the corresponding signaling pathways, which can be effective interventions for the treatment of Cd-induced hepatotoxicity (Yang et al., 2019). QE is a flavonoid rich in antioxidant, antiapoptotic, and anti-inflammatory properties (Anand David et al., 2016). It protects the liver from damage and inhibits its oxidation (Fang et al., 2021). It exerts a protective effect on the toxic damage inflicted by Cd (Prabu et al., 2010), although the mechanisms of protection are not well understood. This study explored the protective effect of QE on Cd-induced BRL-3A cells through the Nrf2 signaling pathway and mitochondrial caspase-dependent apoptosis pathway.

The safe concentrations for CdCl_2_ and QE were determined by measuring the relative survival rate of BRL-3A cells. The IC_50_ of CdCl_2_ was about 50 μmol/L and QE had no inhibitory effect on cell activity. BRL-3A cells were cultured in Cd (12.5 µ mol/L) and QE (5 µ mol/L). It was found that QE improved the cytotoxicity induced by Cd, and the survival rate was significantly higher than that of Cd group. According to reference of Yang et al. (2019), the CdCl_2_ and QE concentrations were finally determined to be 12.5 and 5 μmol/L, respectively.

Elevated levels of liver enzymes represent severe damage to the liver cell membrane, so liver enzymes can be used as markers to assess liver integrity and function (Gong et al., 2019). When the body is healthy, transaminase exists in cells and has little content in serum, when liver cells have pathological changes, the permeability of cell membrane increases, and the transaminase in the cell is released into the blood, resulting in increased transaminase content in the blood (Zhang et al., 2017). LDH is abundant in animal tissues and organs and is released from cells only when the cell membrane is damaged (Jurisic et al., 2015). In the present study, Cd exposure resulted in increased contents of ALT, AST, and LDH medium supernatants, indicating that Cd damaged the hepatocyte membrane.

The main mechanism of Cd-induced liver injury is oxidative stress. Oxidative stress is essentially due to the depletion of antioxidants or the accumulation of ROS, resulting in imbalanced oxidative and antioxidant levels in the body. Cd exposure can directly or indirectly generate excess ROS. Antioxidants in the body such as SOD, CAT, GSH, etc. Constitute the antioxidant system of tissue cells and play antioxidant roles. The existence of oxidase inhibits the oxidative reaction in the body, has the ability to remove ROS and resist oxidative damage, and plays an important role in resisting oxidative damage in animals (Chen et al., 2018). SOD has the function of scavenging oxygen free radicals in the body and SOD levels in organs, which can assess the degree of oxidative damage to organs. CAT breaks down H_2_O_2_ into water and oxygen to reduce oxidative damage to the body. GSH is a potent free-radical scavenger that inhibits ROS generation and is the first line of defense for non-enzymatic antioxidants (NicoleCnubben et al., 2001). The production of MDA indicates the level of cellular damage because MDA is an intermediate product of lipid peroxidation (Luo et al., 2017). In the present study, Cd exposure significantly decreased the SOD and CAT activities and GSH content and significantly increased the MDA content. This finding indicated that Cd exposure led to lipid peroxidation in cells, consumption of antioxidant substances SOD, CAT, and GSH, and oxidative stress in cells.

ROS are highly reactive and can oxidize various biomolecules such as DNA, lipids, and proteins (Cao et al., 2017). ROS can also induce lipid peroxidation, destroy the balance of oxidative and antioxidant systems (Katwal et al., 2018), and disturb the homeostasis of cells and tissues, eventually causing cell damage. Mitochondria are the main targets of Cd-induced apoptosis (Nair et al., 2015; Zhang and Reynolds, 2019), which primarily occurs by activating the mitochondrial apoptosis pathway (Pi et al., 2013; Yin et al., 2018). Cd can directly or indirectly generate excess ROS(Sauvageau and Jumarie, 2013). Sustained and massive ROS production leads to the swelling of mitochondria, rupture of the outer membrane, and decreased ΔΨm (Wang et al., 2020). Moreover, Cd exposure activates the proapoptotic protein Bax (Wang et al., 2020), thereby increasing mitochondrial-membrane permeability, promoting CytC release, triggering a caspase cascade, and leading to apoptosis (Pi et al., 2013; Sandoval-Acuña et al., 2014). In the present study, Cd exposure produced a large amount of ROS, resulting in decreased ΔΨm of BRL-3A cells. The expression levels of Bax mRNA and protein, CytC as well as mRNA and protein, increased. They were released from mitochondria, and the mitochondrial caspase-dependent apoptosis pathway was activated. The expression levels of caspase-9 and caspase-3 mRNA and protein increased, leading to apoptosis. These results were consistent with those of *Paria A* et al. (Amanpour et al., 2020).

Several studies have shown that chronic or acute exposure to Cd can produce large amounts of free radicals, which can lead to oxidative stress in various organs (Patra et al., 2011; Nna et al., 2017). Our findings suggested that Cd induced hepatocyte apoptosis through oxidative stress, so an antioxidant was urgently needed to alleviate the oxidative damage caused by Cd exposure. QE is a natural organic antioxidant (Bahar et al., 2019), It can reportedly counteract Cd-induced neurotoxicity in rat brain, significantly improve Cd-induced abnormalities in biochemical and histological indicators (Prabu et al., 2010), and inhibit Cd-induced autophagy in mouse kidneys (Yuan et al., 2016). In the current work, after QE intervention, the contents of ALT, AST, and LDH in the supernatant of culture medium were reduced, and the damage inflicted by Cd to hepatocytes decreased. We found that QE activated the Cd-inhibited Nrf2 signaling pathway, significantly increased the SOD and CAT activities and the GSH content, significantly decreased the MDA content, reduced ROS generation, and inhibited the expression of the pro-apoptotic proteins Bax, CytC, caspase-9, and caspase-3 mRNA and protein expression. QE also increased the ΔΨm and reduced apoptosis. These results indicated that QE can reduce Cd-induced oxidative stress and apoptosis. The results of. Morales et al. (2006) and. Bu et al. (2011) supported this conclusion.

The Nrf2 signaling pathway and heavy-metal-induced oxidative stress are closely related (Chen et al., 2018; Caiyu Lian et al., 2022), Therefore, the Nrf2 pathway is an important target for preventing Cd-induced liver injury and is a mechanism by which the body resists environmental oxidants (Almeer et al., 2018). Nrf2 is an essential transcription factor whose function is to translocate into the nucleus and interact with the antioxidant response element to promote the transcription of target genes (Yang et al., 2018). Our results showed that Cd exposure inhibited the expression of the Nrf2 signal pathway-related genes and promoted ROS accumulation in cells. After QE intervention, Nrf2 was activated and its entry into the nucleus was promoted, and the expression of Nrf2 nuclear protein increased. Therefore, QE was an effective antioxidant that increased the activity of antioxidant enzymes by activating the Nrf2 signaling pathway, eliminating Cd-induced ROS, alleviating mitochondrial membrane damage, and maintaining the ΔΨm. Consequently, the mitochondrial caspase-dependent apoptosis pathway was inhibited, cell apoptosis was reduced, and the Cd-induced oxidative stress and apoptosis of hepatocytes were alleviated.

## Conclusion

QE exerted an antioxidant effect by activating the Nrf2 signaling pathway in BRL-3A cells, eliminating ROS, and maintaining the integrity of mitochondrial membrane, thereby inhibiting the occurrence of mitochondrial caspase-dependent apoptosis pathway. QE playing a protective role in Cd-induced hepatocyte damage.

## Data Availability

The original contributions presented in the study are included in the article/Supplementary Material, further inquiries can be directed to the corresponding author.
